# Mechanical evaluation for three-dimensional printed orthodontic springs with different heights-*in vitro* study

**DOI:** 10.4317/jced.57908

**Published:** 2021-10-01

**Authors:** Dragan Ströbele, Ahmed Othman, Vasilios Alevizakos, Mesut Turan, Constantin von See

**Affiliations:** 1 Dr. Center of digital technologies in dentistry and CAD/CAM - Danube Private University- Krems- Austria; 2 MSc. Center of digital technologies in dentistry and CAD/CAM - Danube Private University- Krems- Austria; 3 Dr. med. Dent. Center of digital technologies in dentistry and CAD/CAM - Danube Private University- Krems- Austria; 4 Center of digital technologies in dentistry and CAD/CAM - Danube Private University- Krems- Austria; 5 Univ. Prof. Dr. Center of digital technologies in dentistry and CAD/CAM - Danube Private University- Krems- Austria

## Abstract

**Background:**

The orthodontic spring materials in use have a significant influence on the applied forces. The prerequisite to identify the *in vitro*< force deflection of the CAD/CAM fabricated springs is considered mandatory to identify the material characteristics. The purpose of the present investigation was to evaluate the mechanical load on 3D printed springs using different coil heights.

**Material and Methods:**

The springs were digitally designed with different coil heights using Autodesk Netfabb CAD software (San Rafael, CA, USA). Test specimens were manufactured using 3D printable experimental flexible material (Code: BM2008, GC, Tokyo, Japan). The specimens were divided according to the coil height into five groups, group A (n=4mm), group B (n=6mm), group C (n=8mm), group D (n=10mm) and group E (n=12mm). All group specimens were mechanically tested using a universal testing machine. Statistical analysis was performed using K-S-Test to compare the values of each to the control group (*p*< 0.001).

**Results:**

The highest value in all groups was achieved by 5.43 N/mm in group A, while the lowest value was achieved by 0.11 N/mm in group E.

**Conclusions:**

3D printed springs are mechanically affected by the coil heights and there is a direct correlation to the resulting force. Furthermore, the variations within the investigated groups must be thoroughly investigated prior to clinical application.

** Key words:**CAD/CAM, 3D printing, Orthodontics, mechanical testing, material evaluation.

## Introduction

Active orthodontic springs are an accepted method for treating different types of dental malocclusion that can be influenced by genetics, environment and ethnicity (1,2). The importance of orthodontic treatment is its effect to enhance oral functions such as phonetics, mastication, and oro-facial aesthetics. Furthermore, there is an improvement of oral hygienic measures. The treatment of dental malocclusion has its aim in establishment of a balanced sTable occlusion and should be in harmony with facial and aesthetics as well as proper function (3,4). Dental malocclusion can be either treated by fixed or removable functional devices which can include springs (5).

Orthodontic springs are commonly used for space closure, individual tooth retraction or protraction, distal movement of teeth and traction on impacted teeth. Nowadays used material for those springs are stainless steel and nickel titanium (6-8). These materials are in daily use for closing coils that have been used to deliver light and continuous forces in orthodontic tooth movement (9). However, it was found that the forces loss over time is exhibited from most of the used materials (10). The crystalline changes in the martensitic and austenitic phases that occur in the nickel titanium springs materials can affect the resultant forces (11). Even though different materials are used in coil springs additive manufacturing materials have not yet been investigated in depth.

Besides the materials in use the springs design has a significant influence on the applied forces. Thus, different degrees of freedom that can include the helix, loop design or wire diameter result in variable orthodontic forces. The wire thickness and length additionally are variables influencing the amount of forces acting upon the tooth as well as its direction (12,13).

The force-displacement plots upon spring activation and loading until maximum possible reached force had been *in vitro* and *in vivo* examined for the nickel titanium springs (14).

Different studies were found comparing the mechanical properties of the nickel titanium and commercially used springs (15). The prerequisite to identify the *in vitro* force deflection of the CAD/CAM fabricated springs is considered mandatory to identify the material characteristics. Also, the digitally manufactured springs can be custom designed according to each case independently. Accordingly, mechanical systematical testing for CAD/CAM orthodontic springs using 3D printed resin *in vitro* is mandatory prior to clinical procedures.

## Material and Methods

-Material

A total of five groups were used for this study, with 10 specimens in each group. 3D orthodontic springs using Max 3D printer (Asiga, Sydney, Australia) via digital light processing technology with the experimental flexible material (Code: BM22008, GC, Tokyo, Japan) were printed. Different coil height parameter ranging from 4 to 12mm, were used in this study with 8mm as control group. All groups with 4 coils was selected referring to a study conducted by Othman *et al*. who found that comparing between the conventional laboratory fabricated springs with the CAD/CAM methods had a significant difference for material as well as design comparing compression to force ratio (16).

**Figure 1:**
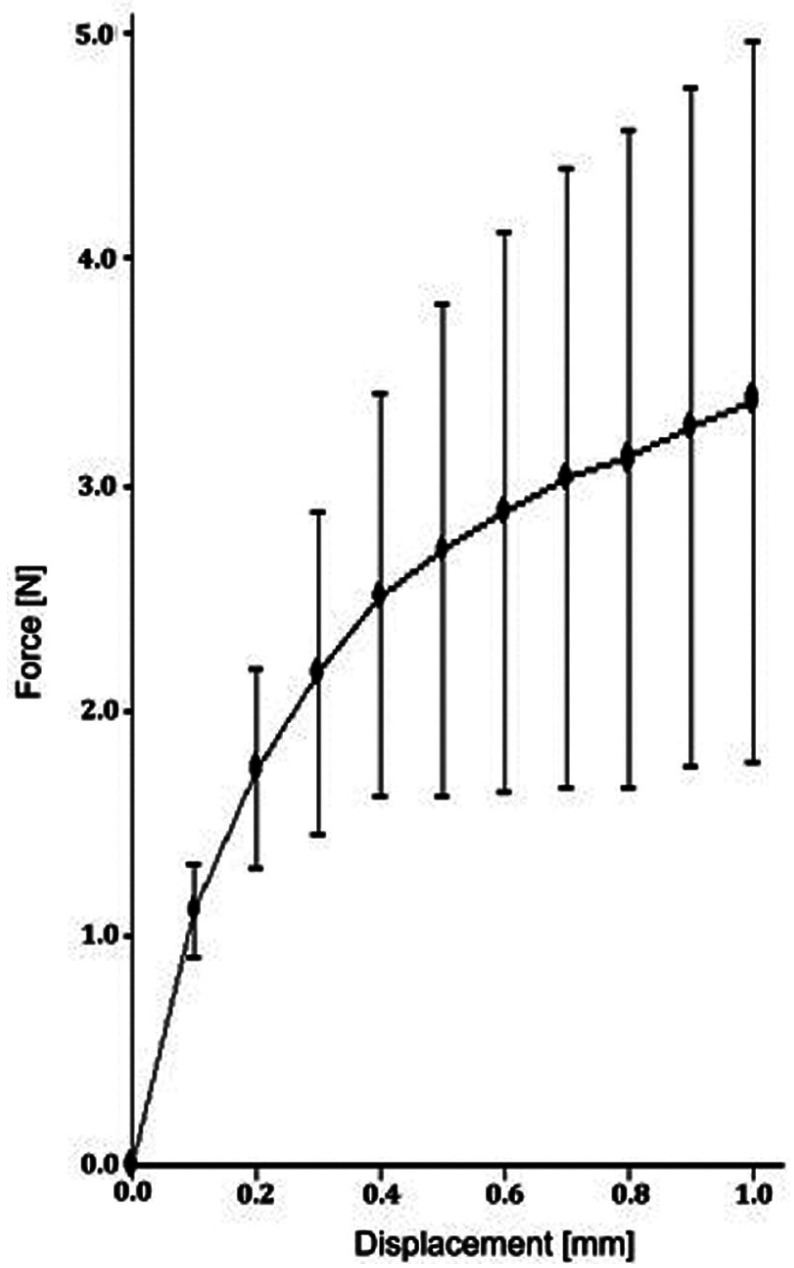
Impact of applied force on the displacement of the spring (A).

The five groups were alphabetically numerated into group A (4mm), group B (6mm), group C (8mm), group D (10mm) and group E (12mm). All specimens were digitally designed using Autodesk Netfabb (San Rafael, CA, USA) with the same radius and diameter for all test specimen. Post processing of the specimen was performed according to the manufacturer’s instructions. Therefore, unheated ultrasonic reusable isopropanol solution with the concentration 96% was used to clean the specimens for 2 minutes followed by 2 more minutes of a clean isopropanol bath with same concentration. All specimens were withdrawn from the solution bath and dried with compressed air in-between the two cleaning cycles. Surface polymerization was done using Labolight DUO (GC, Tokyo, Japan) with double wave length LED technology in a range of 380nm – 510nm with spectrum ranges peaks of 465nm - 485nm (12 Blue LED’s) and 390nm - 400nm (3 Violet LED’s) for three minutes from both sides. After curing, carbide bur and nipper were used to remove the supports (Table 1) (Fig. 1).

**Table 1:**
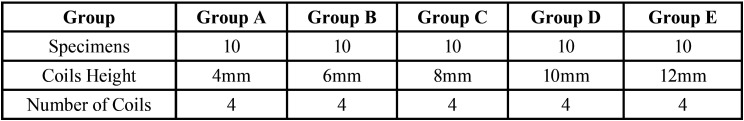
Overview of the group classification.

-Method

All specimens were mechanically tested to determine the compression properties. Each specimen was mounted into a universal testing machine (Z010 Zwick, Ulm, Germany) for compression testing. The overall testing time was mechanically tested for 50 minutes, undergoing 10 cycles of loading. The total movement distance of the piston machine per testing was 1mm (0.1mm per cycle).

For establishing comparable testing circumstances, support attachments for the springs were used, which enable reproducible positioning of the specimens in the universal testing machine.

The machine output provides force/position data over time which was stored and analyzed statistically offline.

-Statistical analysis

Statistical analysis including mean and SD were calculated for each sample at each compression interval over time. These data were analysed comparing each group for the given movement/compression using Sigma software (Sigma Plot 13, Systat Software Inc., USA). Statistical analysis was performed using K-S-Test to compare mean values of the groups with the control group. Significance level was defined as *p* <0.05.

## Results

In group A, the highest value was reached with a mean force of 3.48N ± 1.53 for 1.0mm compression, while the lowest value was reached in group E with a mean force of 0.25N ± 0.12 for 1.0mm compression. A regression for the mean as well as maximum and minimum decreased for the spring heights to compression force (Table 2).

**Table 2:**
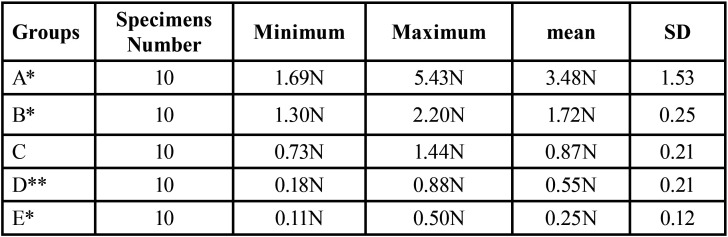
Statistical analysis of the measured forces according to group A-E.

**Figure 2:**
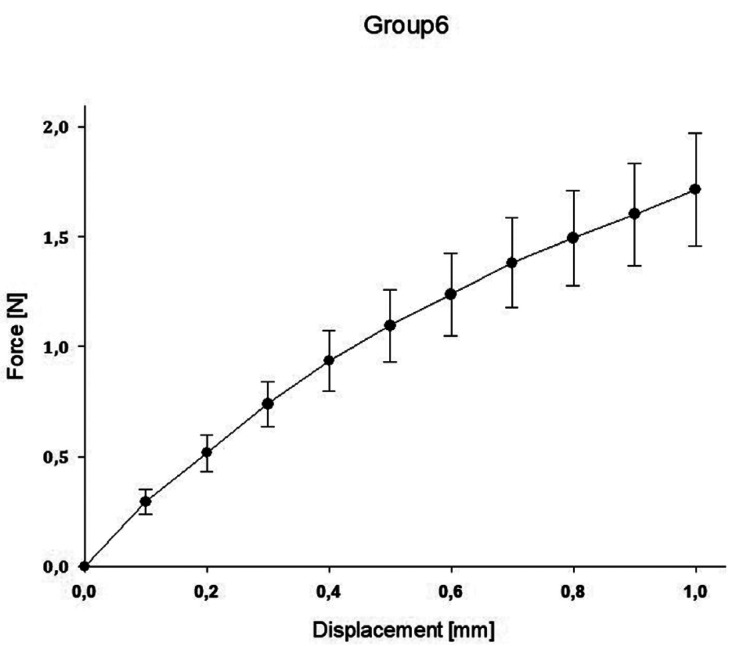
Impact of applied force on the displacement of the spring (B).

**Figure 3:**
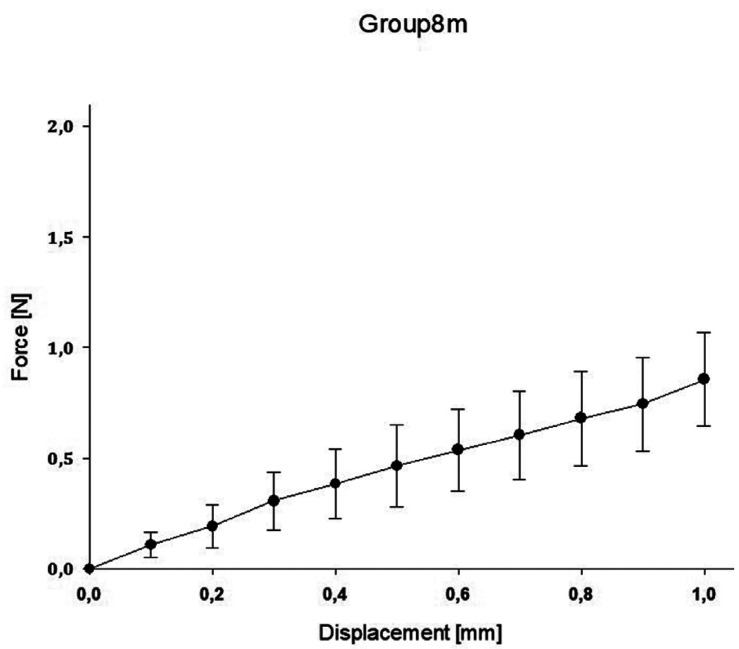
Impact of applied force on the displacement of the spring (C).

**Figure 4:**
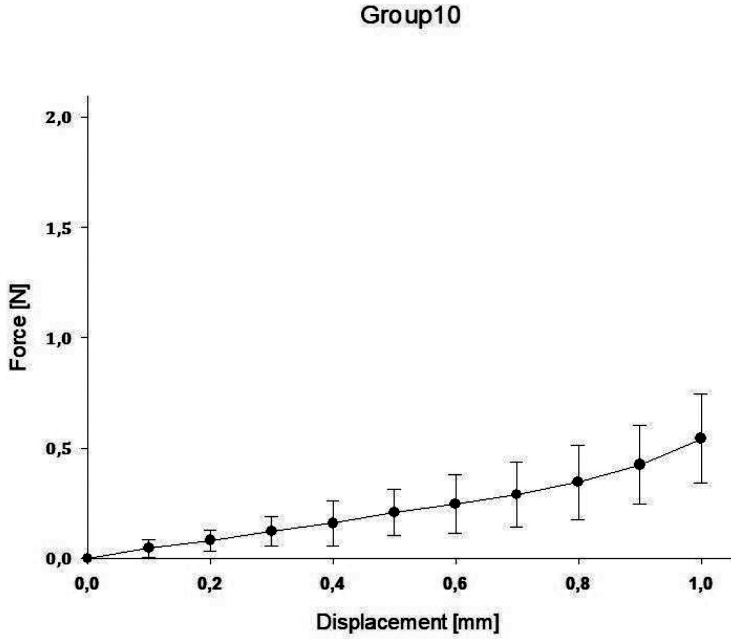
Impact of applied force on the displacement of the spring (D).

**Figure 5:**
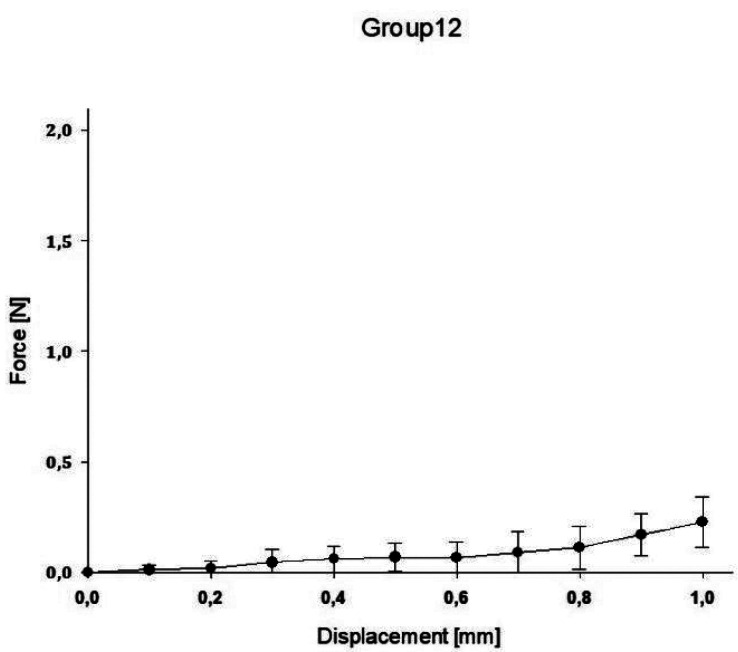
Impact of applied force on the displacement of the spring (E).

The impact of the applied force on the displacement of the springs is displaced for all groups in the following Figures (Figs. 1-5).

## Discussion

The inconsistency to obtain a predicTable compression forces using 3D printed orthodontic springs is a new issue of concern in digital orthodontics. The specific properties of this material and its deviation are unknown.

In this *in-vitro* study 3D printed springs with different coil heights were investigated regarding their mechanical properties. The results show that springs with lower coil’s height reached statistically significantly higher values of compression load than springs with higher ones.

Concerning materials and methods, all specimens were 3D designed (Auto desk Netfabb, San Rafael, CA, USA) and 3D printed (MAX Asiga, Sydney, Australia). Nowadays 3D printers such as Digital Light Processing, Selective Laser Sintering (SLS), Stereolithography (SLA) and Fused Deposition Modelling (FDM) are common (17). Printing materials of FDM/FFF are composed of acrylonitrile butadiene styrene and polylactic acid, whereas DLP, SLA and SLS consist of photopolymer (18). Regarding the precision of common printing methods Kim SY *et al*. conducted that DLP technique is more precise than FFF and SLA technique (19). Also, Zang *et al*. conducted in a recent investigation, that DLP technique is more precise at increasing layer thickness than SLA technique (20). In this investigation digital light processing (DLP) was used.

The study protocol followed the manufacturer’s instructions for the post processing to ensure uniform results. Using mechanical testing machine Z010 (Zwick/Roell, Ulm, Germany) for investigating the force of a spring related to its height. The spring design was maintained by same number of coils, diameter and helix. However, the height design was variable.

Clinical usage cannot be entirely *in vitro* simulated within standardized test, however material specific properties can be found. With the objective to indicate which orthodontic spring height mostly affects the resulting force, a comparison of all groups with control group C was conducted. Significant difference between all groups compared to group C was proved. Regarding the maximum value reached by group A of 5.43N and the other groups, the values in group A were highly scattered. However, all other groups showed more sTable values in the force/deflection graph. The control group exhibited the most sTable value during mechanical testing.

Representative for the experiment was proportionality of height changing and force loading. A greater height design resulted in less loading force of the spring. For clinical use a constant force is necessary to obtain an optimum orthodontic tooth movement (21). In order to achieve different tooth movements, such as tipping, translation and rotation, forces ranging up to 300g are required (22). From the point of this experiment compression forces of more than 300g were achieved, caused by varying heights of the spring design.

## Conclusions

This current study demonstrated that the CAD/CAM fabricated orthodontic springs could produce relevant forces that are variable according to the design parameter. The practitioner can determine the appropriate force, in order to accomplish optimum tooth position. Spring height 6mm and 12mm are mostly mechanically affected. Respectively Group B and E are the highly significant values in this investigation (*P*<0.001). Leaning at this *in vitro* study, there is a possibility for further studies for possible usage of 3D fabricated orthodontic springs.
